# Cancer immunotherapy with γδ T cells: many paths ahead of us

**DOI:** 10.1038/s41423-020-0504-x

**Published:** 2020-07-22

**Authors:** Dieter Kabelitz, Ruben Serrano, Léonce Kouakanou, Christian Peters, Shirin Kalyan

**Affiliations:** 1 Institute of Immunology, Christian-Albrechts University of Kiel and University Hospital Schleswig-Holstein Campus Kiel, D-24105 Kiel, Germany; 2 Faculty of Medicine, University of British Columbia, Vancouver, BC V6T 1Z4 Canada

**Keywords:** Adoptive T cell transfer, Antibody constructs, Cytokines, gamma/delta T cells, Immunotherapy, Leukemia, Lymphoma, Solid tumors, Immunosurveillance, Immunology

## Abstract

γδ T cells play uniquely important roles in stress surveillance and immunity for infections and carcinogenesis. Human γδ T cells recognize and kill transformed cells independently of human leukocyte antigen (HLA) restriction, which is an essential feature of conventional αβ T cells. Vγ9Vδ2 γδ T cells, which prevail in the peripheral blood of healthy adults, are activated by microbial or endogenous tumor-derived pyrophosphates by a mechanism dependent on butyrophilin molecules. γδ T cells expressing other T cell receptor variable genes, notably Vδ1, are more abundant in mucosal tissue. In addition to the T cell receptor, γδ T cells usually express activating natural killer (NK) receptors, such as NKp30, NKp44, or NKG2D which binds to stress-inducible surface molecules that are absent on healthy cells but are frequently expressed on malignant cells. Therefore, γδ T cells are endowed with at least two independent recognition systems to sense tumor cells and to initiate anticancer effector mechanisms, including cytokine production and cytotoxicity. In view of their HLA-independent potent antitumor activity, there has been increasing interest in translating the unique potential of γδ T cells into innovative cellular cancer immunotherapies. Here, we discuss recent developments to enhance the efficacy of γδ T cell-based immunotherapy. This includes strategies for in vivo activation and tumor-targeting of γδ T cells, the optimization of in vitro expansion protocols, and the development of gene-modified γδ T cells. It is equally important to consider potential synergisms with other therapeutic strategies, notably checkpoint inhibitors, chemotherapy, or the (local) activation of innate immunity.

## Introduction

γδ T cells comprise a relatively small subset of T lymphocytes in the peripheral blood of adult individuals. While there is substantial interindividual variability, γδ T cells usually account for anywhere between 1 and 10% of CD3^+^ T cells in human blood,^1^ and there are age-dependent alterations in the proportion and T cell receptor (TCR) repertoire of γδ T cells in the blood.^1,2^ γδ T cells are more abundant at barrier sites such as the intestine; up to 20% of intraepithelial CD3^+^ T cells in the human colon express the γδ TCR.^3^ Interestingly, there are significant species-specific differences in the abundancy of γδ T cells. As an example, much higher numbers of γδ T cells are present in the blood of ruminants than in the blood of humans.^4^ In contrast to conventional T cells bearing an αβ TCR that recognizes antigen-derived peptides loaded onto MHC molecules (human leukocyte antigen [HLA] in humans), γδ T cells typically recognize their ligands independent of antigen processing and MHC/HLA restriction.^5^ The dominant population of γδ T cells in the blood of healthy adults expresses a TCR composed of the variable (V) gene Vγ9 paired with Vδ2. Such Vγ9Vδ2T cells (for simplicity referred to as Vδ2 in the following sections) account for anywhere from 50 to more than 95% of peripheral blood γδ T cells, with remarkable donor-dependent variability.^6,7^ The second most frequent γδ T cell subset in blood expresses the variable Vδ1 chain, which can be paired with any of the six expressed Vγ genes. Importantly, such Vδ1T cells (and other non-Vδ2 γδ T cells, mostly Vδ3) are more abundant in the intestinal mucosa,^8^ in line with differential ligand recognition of peripheral blood and mucosal γδ T cells.^5,9^ γδ T cells are considered to have their niche at the crossroad of innate and adaptive immunity.^9^ They share features of the adaptive immune system, with their expression of clonally rearranged TCR genes, but at the same time are similar to innate immune cells, with the lack of need for antigen processing to activate their effector functions. Therefore, γδ T cells rapidly respond to TCR triggering. Moreover, γδ T cells frequently coexpress functional receptors of innate immune cells, such as activating natural killer (NK) receptors such as NKG2D, NKp30, and/or NKp44, which directly trigger cytotoxic activity,^10–13^ in addition to certain Toll-like receptors (TLRs), which can provide costimulatory signals.^14,15^ At the level of effector activity, γδ T cells share many functions with αβ T cells. Activated γδ T cells have the capacity to be potent killers that can lyse a broad variety of solid tumor and leukemia/lymphoma cells and produce an array of cytokines.^16–19^ Depending on the local micromilieu, γδ T cells can differentiate into Th1-, Th2-, Th9- or Th17-like cells and produce prototypical cytokines such as interferon-γ (IFNγ) and interleukin (IL)-4/-10, IL-9, or IL-17.^20–23^

## Ligand recognition by human γδ T cells

**Fig. 1:**
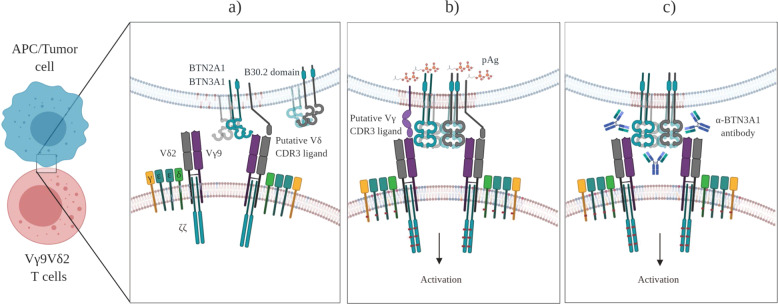
Role of BTN2A1 and BTN3A1 in the activation of human Vγ9Vδ2 γδ T cells. The butyrophilin members BTN2A1 and BTN3A1 are loosely associated on the surface of target cells. **a** In the homeostatic “resting” state, the intracellular B30.2 signaling domain does not associate with endogenous (tumor-derived IPP) or exogenous (microbe-derived HMBPP) phosphoantigens (pAgs). However, BTN2A1 binds to germ-line-encoded regions of the Vγ9 chain in the homeostatic state. There is also evidence that the CDR3 region of the TCR δ chain interacts with another currently unidentified ligand.^37^ **b** In infected cells and tumor cells, exogenous (HMBPP) or endogenous (IPP) pAgs bind to the B30.2 domain and thereby induces a conformational change in the BTN2A1–BTN3A1 complex, resulting in TCR-dependent activation of Vγ9Vδ2 T cells. This step may involve other as yet unidentified CDR3 ligands.^36,37^ **c** Agonistic anti-BTN3A antibodies such as clone 20.1 mimic the activity of pAgs by inducing a conformational change in the BTN molecules, leading to γδ T cell activation. The depicted model is based on refs. ^35–38^

Although γδ T cells were discovered in the mid-1980s, the nature of the antigens recognized by the γδ TCR has largely remained a mystery that we continue to dissect. Today, some antigens with relevance for immune surveillance by specific subsets of γδ T cells have been well characterized. The most conspicuous ligands for human Vδ2T cells are small pyrophosphate molecules, which are intermediates of the cholesterol synthesis pathway in microbes and eukaryotic cells. Many bacteria and some parasites use the so-called non-mevalonate (or Rohmer) pathway of isoprenoid biosynthesis, of which (*E*)-4-hydroxy-3-methyl-but-2-enyl pyrophosphate (HMBPP) is an intermediate that exclusively activates Vδ2T cells at picomolar to nanomolar concentrations.^24,25^ The synthetic molecule bromohydrin pyrophosphate (BrHPP) is similarly active at nanomolar concentrations.^26^ Homologous pyrophosphate molecules, specifically isopentenyl pyrophosphate (IPP), are also generated in the mevalonate pathway of cholesterol synthesis in eukaryotic cells. However, much higher concentrations (in the micromolar range) of IPP are required to activate the same population of Vδ2T cells. While normal cells do not accumulate sufficient IPP to activate γδ T cells, many transformed cells have a dysregulated mevalonate pathway, leading to increased IPP accumulation and consequent γδ T cell activation.^27–30^ Importantly, the activation of human Vδ2T cells by such phosphoantigens is crucially dependent on transmembrane butyrophilin (BTN) molecules. The landmark study by Harly and coworkers has identified BTN3A1 and its intracellular B30.2 signaling domain as being indispensable for the activation of Vδ2T cells by phosphoantigens.^31^ Subsequently, detailed molecular studies have shown that pyrophosphates directly bind to the intracellular B30.2 domains and trigger inside-out signaling to activate Vδ2T cells.^32,33^ This process is modulated by the GTPase Rho in tumor cells, which is recruited to the B30.2 domain, thereby inducing changes in the cytoskeleton, as well as conformational changes in BTN3A1.^34^ However, very recent studies from three independent laboratories indicate that another member of the BTN family is also required. Using different experimental approaches, it was found that BTN2A1 collaborates with BTN3A1 in sensitizing pAg-exposed cells to recognition by human γδ T cells. While BTN2A1 directly binds to the TCR via germline-encoded regions of the Vγ9 chain, it is suggested that, following pAg binding to the B30.2 domain, the BTN2A1–BTN3A1 complex engages additional regions of the TCR. These exciting new results also indicate that there might be the recruitment of an additional as yet unidentified CDR3 ligand upon complex formation of BTN2A1, BTN3A1, and pAgs.^35,36^ Recent results from J. Kuball’s group have added greater complexity to the molecular mechanisms involved in BTN-dependent tumor recognition by Vδ2T cells. According to these studies, the binding of TCRγ regions between CDR2 and CDR3 to BTN2A1 is followed by the binding of the TCRδ CDR3 to an as yet unidentified ligand. This process is pAg independent. Full activation of the γδ TCR requires pAg- and RhoB-dependent recruitment of BTN3A1 (together with BTN2A1) to the immunological synapse.^37^ In any case, it is clear that pAg accumulation in transformed cells is critically important for BTN-dependent activation of tumor-reactive Vγ9Vδ2T cells. Interestingly, BTN-dependent selective activation of Vγ9Vδ2T cells can also be achieved with agonistic anti-BTN3A1 (CD277) monoclonal antibodies (mAbs). Agonistic antibodies (e.g., clone 20.1) mimic pAg-dependent activation, whereas antagonistic mAbs (e.g., clone 103.2) inhibit the activation of Vγ9Vδ2T cells.^25,31^ Importantly, recent insights revealed that the agonistic activity of mAb 20.1 also depended on cell surface-expressed BTN2A1.^35^ The currently discussed model^38^ of Vγδ2 T cell activation and the role of BTN molecules and pAgs are illustrated in Fig. 1.

There is no doubt that the respective roles of pyrophosphate antigens and BTN2A1/BTN3A1 are the best understood mechanisms of activation requirements of a specific population of human γδ T cells. Additional reported ligands for the Vγ9Vδ2 subset include the complex of cell surface F1-ATPase and apolipoprotein A-I^39^ and the ectopically expressed DNA mismatch repair protein human MutS homologue 2 (hMSH2).^40,41^ The knowledge of specific ligands for other γδ T cell subsets is less advanced, but there are several interesting findings that have been reported that are worth noting. Early studies indicated that non-Vδ2 (mainly Vδ1 and Vδ3) γδ T cells were expanded in the blood of kidney transplant recipients who experienced cytomegalovirus (CMV) infection after transplantation.^42^ Subsequently, it was shown that such CMV-reactive non-Vδ2 γδ T cells also recognized various intestinal epithelial tumor cells.^43^ The endothelial protein C receptor (EPCR) expressed on CMV-infected endothelial cells, which is also aberrantly expressed on epithelial tumor cells, has been identified as a specific ligand for human Vγδ5 γδ T cells.^44^ Along the same line, the intracytoplasmic phospholipid binding protein Annexin A2 (Anx A2) translocates to the cell surface following exposure to oxidative stress. Anx A2 expressed on the surface of tumor cells is reported to be a ligand for certain Vδ2-negative, specifically Vγ8Vδ3, γδ T cells.^45^ Moreover, human intestinal Vγ4 γδ T cells coexpressing Vδ1 or Vδ3 were recently shown to recognize butyrophilin-like proteins BTNL-3 and BTNL-8 in a TCR-dependent manner on intestinal epithelial cells.^46^ In these instances, BTNL responsiveness is mediated by germline-encoded motifs within the Vγ4 chain.^47,48^ In addition, human Vδ1-expressing γδ T cells can recognize microbial and self-lipids bound to the nonclassical MHC protein CD1d.^49^ While it is to be expected that more specific ligands for various γδ T cell subsets will be identified in the future, the emerging overall picture clearly indicates that the TCR of γδ T cells is constantly being scrutinized for signs of “stress” on normal cells and those undergoing malignant transformation, thereby assigning γδ T cells an important place in local immune surveillance.^50,51^ This conclusion is well supported by a pivotal study demonstrating an increased susceptibility to tumor development in γδ T cell deficient mice.^52^

**Fig. 2:**
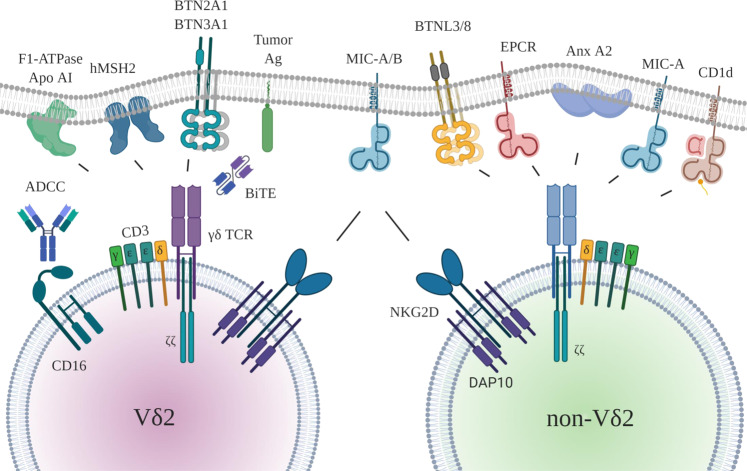
Major receptor–ligand interactions between Vδ2/non-Vδ2 γδ T cells and tumor cells/antigen-presenting cells. Left side*:* The best characterized ligands for the human Vγ9Vδ2 TCR are phosphoantigens (pAgs), which are recognized in a BTN2A1-/BTN3A1-dependent manner. Other ligands for this TCR include the ectopically expressed DNA repair protein human MutS homologue 2 (hMSH2) and ectopically expressed F1-ATPase in conjunction with apolipoprotein A-I. Tumor antigen–TCR crosslinking bispecific T cell engagers (BiTEs) also activate Vδ2 T cells via the TCR. Therapeutically used antibodies against tumor (associated) antigens can activate γδ T cells via CD16/FcγRIII-dependent ADCC. Right side*:* Ligands for non-Vδ2 γδ T cells. Some ligands for specific subsets of human non-Vδ2 γδ T cells have been identified: endothelial protein C receptor (EPCR) on CMV-infected and intestinal tumor cells (Vγ4Vδ5), butyrophilin-like molecules BTNL3/BTNL8 on intestinal epithelia (Vγ4/Vδ1 or Vδ3), Annexin A2 (Anx A2) (Vγ8Vδ3), MICA (Vδ1), and lipids bound to CD1d (Vδ1). MICA/B and ULBP molecules are ligands for the activating NKG2D receptor expressed on both Vδ2 and non-Vδ γδ T cells. In addition to NKG2D, other NK receptors (NKp30, Nkp44, NKp46) can be expressed as well

In addition to TCRs, γδ T cells usually express other activating receptors. As already mentioned, most human γδ T cells also carry the NKG2D receptor on the cell surface, which recognizes stress-inducible MHC class-I-related molecules frequently expressed on transformed cells but absent on normal cells. The NKG2D receptor also contributes to immune surveillance, as illustrated by increased tumor incidence in NKG2D-deficient mice.^53^ Most solid tumors as well as leukemias express at least one of the eight NKG2D ligands (MHC class I-related chain A/B [MICA/B], UL16-binding proteins [ULBP1–6]),^54^ and the cytotoxic effector function of γδ T cells can be triggered through NKG2D/NKG2D ligand interactions independent of TCR signaling.^16,55^ Notably, however, NKG2D ligands are not uniformly expressed on malignant cells. It appears that leukemia stem cells may lack NKG2D ligand expression,^56^ and this absence of expression can render them less susceptible to γδ T cell recognition. As an exhibit of validating redundancy, it has been found that the NKG2D ligand MICA can also be recognized by the Vδ1 TCR,^57^ and direct binding of MICA to Vδ1 has been demonstrated.^58^ Moreover, other NK receptors, such as NKp30, NKp44, and DNAM-1 (CD226), can also be expressed at varying levels on γδ T cells and contribute to tumor cell recognition and killing.^12^ Recently, NKp46 was shown to be specifically expressed on gut-resident intraepithelial human Vδ1 T cells endowed with potent antitumor activity.^59^ Taken together, γδ T cells are uniquely equipped with two independent recognition pathways to sense stressed and transformed cells, i.e., TCRs as well as activating NK receptors.^12,16^ A schematic overview of the major receptor–ligand interactions involved in the activation of human Vδ2 and non-Vδ2 γδ T cells is shown in Fig. 2.

## Tumor-infiltrating γδ T cells: friends or foes?

Many studies with in vitro activated cells isolated from peripheral blood have demonstrated potent and HLA-independent activity of γδ T cells against various solid tumors and leukemia/lymphoma cells. γδ T cells can also infiltrate tumors where they exert protumorigenic activities or contribute to tumor regression. What information can then be gathered from the analysis of tumor-associated γδ T cells, and how does this correlate with prognostic significance? There are three ways of approaching this question: (i) transcriptional analysis of bulk tumor transcriptomes in large cohorts of patients; (ii) immunohistological characterization of tumor-infiltrating γδ T cells in the context of the in situ tumor micromilieu; and (iii) phenotypic and functional studies of tumor-infiltrating γδ T cells.

**Fig. 3:**
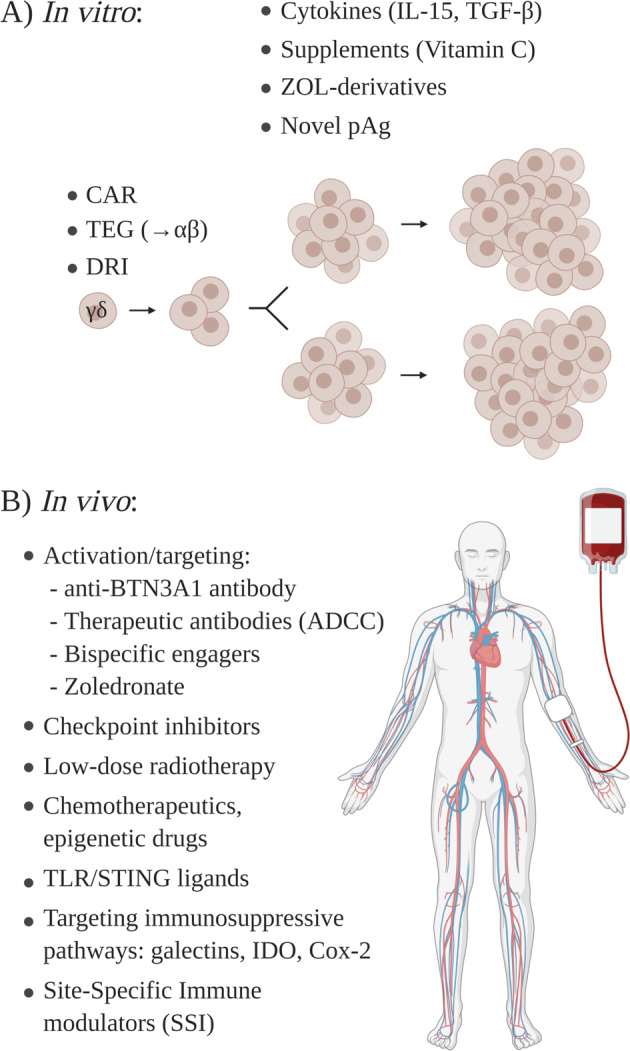
How to enhance the efficacy of γδ T cell immunotherapy in vitro and in vivo. **a** Genetic engineering is used to transduce chimeric antigen receptors (CARs) into γδ T cells, to transfect αβ T cells with selected high affinity γδ TCRs (TEGs), or to render γδ T cells drug-resistant (drug-resistant immunotherapy, DRI). In vitro expansion of γδ T cells can be optimized by selected cytokines (e.g., IL-15, TGF-β), specific medium supplements (e.g., vitamin C), or the selection of novel activators (ZOL derivatives, novel pAgs). **b** Multiple strategies are available to activate γδ T cells in vivo, to target γδ T cells to tumor antigens, to target tumor-intrinsic suppressive pathways, or to increase the local inflammatory milieu

Analyzing transcriptomes from 18,000 tumor samples across 39 different cancer types using the CIBERSORT algorithm,^60^ Gentles et al. identified the abundance of γδ T cells as the single most favorable prognostic parameter out of 22 distinct leukocyte subsets.^61^ Figure 3c from their paper is frequently presented to support the notion that γδ T cells are critical for optimal tumor defense. Technical limitations of this approach were later noted, as it did not appropriately differentiate between γδ T cells and other T cell subsets and NK cells.^62^ Tosolini and coworkers improved the computational CIBERSORT identification of tumor-infiltrating Vγ9Vδ2T cells by the deconvolution of cancer microarray data sets using machine-learning methods, revealing more variability with respect to interindividual variation and the respective cancer type. Overall, the abundance of Vγ9Vδ2 tumor-infiltrating T cells in this study was associated with a favorable outcome in colorectal carcinoma, prostate carcinoma, chronic lymphocytic leukemia (CLL) and acute myeloblastic leukemia (AML).^62^ Several other tools have been developed to monitor T cell subset abundance from RNAseq and microarray expression data in cancer patients. ImmuCellAI has been recently introduced for estimating 18 T cell subsets, including γδ T cells. The method has been validated with flow cytometry results and was shown to allow predictions for immunotherapy responses.^63^ As an example, this algorithm revealed that γδ T cell infiltration was significantly greater in responders than in nonresponders in 58 melanoma samples from a clinical trial with anti-PD1 checkpoint inhibitor therapy.^63^ On the other hand, it was observed in a recent CIBERSORT-based analysis that the abundance of γδ T cells is associated with poor prognosis in pancreatic adenocarcinoma. Together with M0 macrophages and naïve CD4 T cells, γδ T cells contributed to an immune score that was superior to the classic TNM staging.^64^

In addition to whole-tissue transcriptomic analysis and phenotypic/functional characterization of isolated tumor-infiltrating lymphocytes (TILs), immunohistochemistry can provide important insights into the localization of γδ T cells within tumors and the surrounding tissue. Such investigations (usually combined with functional analysis of isolated TILs) have been performed in several types of cancer, including melanoma, pancreatic adenocarcinoma, glioblastoma multiforme, colorectal carcinoma, hepatocellular carcinoma, breast cancer, and others.^65–70^ In some studies, γδ T cells comprised up to 20% of CD3^+^ TILs.^68^ Due to the limited availability of antibodies suitable for immunohistological analysis of paraffin-embedded tissue or frozen sections, most studies have focused on the detection of all γδ T cells using a pan-TCRγδ mAb. In several instances, it was observed that γδ T cells are localized in the periphery of the tumor or in peritumoral tissue, suggesting that strategies to enhance tumor infiltration might improve the antitumor activity of γδ T cells.^65,68,69^ A very recent study by Chabab and colleagues established a standardized protocol to analyze γδ T cells in tumor tissue microarrays using the pan-TCRγδ mAb clone H-41. They applied this approach to quantify γδ TILs in breast, colorectal, pancreatic and ovarian cancer, demonstrating the variability of γδ T cell infiltration in different tumor entities.^71^ Further optimization of staining protocols and antibody clone selection, which includes suitable mAbs directed against γδ T cell subsets, will help to improve immunohistological studies in the future.^72^ We anticipate that interest in γδ T cell subset analysis within tumors in situ will be invigorated with innovative technologies such as fully automated high-content imaging and quantitative whole-slide imaging analysis.^73,74^

Phenotypic and functional characterization of γδ T cells within TILs freshly isolated from various tumors has been extensively performed. A comparison between γδ T cells within the TILs and peripheral blood of the same patient frequently revealed an altered γδ T cell subset distribution across different tumor entities, with higher proportions of Vδ1T cells being present in the tumor than in the blood.^68,75–77^ Of particular interest is the functional analysis of γδ TILs and their TCR repertoire in comparison to blood to determine whether there is preferential recruitment of clonal γδ T cells to the tumor site. In different tumors, γδ TILs frequently produce IFN-γ,^68,77^ and no major difference was observed in this respect between Vδ1 and Vδ2 TILs in an ovarian cancer study.^77^ Furthermore, most, if not all, Vδ1 and Vδ2 TILs produced granzyme A and B, in line with their cytotoxic capacity.^77^ In several experimental settings, IL-17-producing γδ T cells have been implicated in tumorigenesis and metastasis formation through the recruitment of tumor-promoting macrophages and neutrophils^78–80^ or the induction of angiogenesis in response to human papilloma virus (HPV)-16 oncoprotein expression.^81^ In a recent study using conventional and germ-free mice, it was observed that commensal microbiota present in the lung can indirectly stimulate IL-17 production in lung-resident γδ T cells, thereby promoting inflammation and lung cancer development.^82^ There are multiple pathways through which IL-17 can promote tumorigenesis, including effects on angiogenesis, endothelial cell permeability, or the upregulation of adhesion molecules.^83^ It should be noted that the capacity for IL-17 production is much lower for human γδ T cells than for murine γδ T cells. Nevertheless, IL-17-producing cells among γδ TILs (usually of the Vδ1 subgroup) have been identified in several different tumors, and tumor-promoting activity has been suggested.^84–86^ However, IL-17 production is not a general feature of human γδ TILs. While both Vδ1 and Vδ2 TILs in ovarian cancer produced IFN-γ and granzyme A/B, very little, if any, IL-17 was detected.^77^ Moreover, there is controversy among published studies in the same tumor entity. Wu et al. detected high proportions of IL-17-producing cells among γδ TILs in colorectal cancer,^84^ whereas Meraviglia et al. reported only low numbers in a different cohort of colorectal cancer patients.^68^ Very few IL-17-expressing γδ T cells among breast-cancer-infiltrating γδ TILs were reported in a recent study by Janssen et al.^87^ Again, we expect that better conclusions can be drawn in future studies using automated high-content imaging of tumor tissue to more precisely define and quantify immune cell composition within the tumor and peritumoral tissue. As yet, limited information is available on the TCR repertoire of γδ TILs in human tumors. Based on antibody staining with available mAbs to identify expressed TCR Vγ chains,^72^ an increase in non-Vδ2 γδ T cells coexpressing Vγ2/3/4 among ascites and TIL γδ T cells compared to levels in peripheral blood was observed in ovarian cancer patients.^77^ In glioblastoma multiforme, unique TCR clonotypes were identified by next-generation sequencing in intratumoral Vγ9Vδ2 γδ T cells compared to peripheral blood, suggesting specific recruitment of selected γδ T cells to the tumor site.^67^ Furthermore, a distinct TCR repertoire was identified in gut-resident Vδ1 γδ T cells that expressed NKp46 and had high cytotoxic potential against colorectal cancer.^59^ The γδ TCR repertoire has been extensively studied during normal development and in infectious diseases.^88^ Some studies have also noted different γδ TCR repertoires in the blood of cancer patients than in healthy controls.^89^ However, to better understand the significance of clonal γδ T cell recruitment to the tumor site, more equivalent studies in various tumors and lymphomas are needed.

Vδ1 and Vδ2 γδ T cells exerting potent antitumor cytotoxic activity can be readily expanded from peripheral blood and ascites,^18,90–100^ despite the potential protumorigenic activity of γδ T cells residing in situ within the tumor. The mechanisms by which intratumoral γδ T cells might in fact promote tumorigenesis and metastasis formation have been recently reviewed in several excellent articles.^83,101,102^ Depending on the cancer type, such mechanisms might include various effects, such as the recruitment of myeloid-derived suppressor cells (MDSCs) by IL-17-secreting γδ T cells^84^ or the suppression of αβ T cell responses by tumor-infiltrating γδ T cells mediated by PD-L1-dependent mechanisms,^103^ TLR8-dependent pathways^104^ or CD73.^105^ However, again, conflicting observations about the role of specific γδ T cell subsets in a given tumor type have been reported. Vδ1 TILs in breast cancer were shown to suppress αβ T cells,^104^ whereas a recent study actually identified innate-like Vδ1 TILs as being associated with remission in triple-negative breast cancer.^106^ We conclude that the protumorigenic potential of γδ T cells must be well taken into account; however, there is a substantial body of evidence supporting the notion that γδ T cells are exceptional candidates for cellular immunotherapy.

## γδ T cells: regulating and being regulated

γδ T cells to be applied for cancer immunotherapy should not exert suppressive activity. To ensure this, it is important to understand which signals can impose regulatory functions on γδ T cells. Transforming growth factor-β (TGF-β) is well known to induce a regulatory phenotype for CD4 T cells (iTregs).^107^ Casetti et al. first showed that TGF-β can induce FOXP3, the master transcription factor of Treg, and regulatory activity in human Vδ2T cells.^108^ The suppressive activity of Tregs critically depends on the demethylation of Treg-specific demethylated regions (TSDRs) in the *FOXP3* gene.^109,110^ Vitamin C (Vit C) is a well-characterized cofactor for the activation of ten-eleven translocation (Tet) enzymes that mediate DNA hydroxymethylation, including the hypomethylation of *FOXP3* TSDRs.^111^ We recently studied the effect of Vit C on TGF-β-induced regulatory activity and FOXP3 expression in human Vδ2T cells. We observed a strong enhancement of FOXP3 expression and regulatory activity of purified γδ T cells stimulated with phosphoantigen and TGF-β in the presence of Vit C. More importantly, strong hypomethylation of *FOXP3* TSDRs was observed only in the presence of Vit C, suggesting that TGF-β frequently expressed in the tumor microenvironment might prime local γδ T cells for suppressive activity if additional epigenetically active signals are present.^112^ In some circumstances, however, it appears that γδ T cells can also downregulate αβ T cell responses independent of FOXP3 expression. Upon activation, γδ T cells transiently upregulate various inhibitory receptors and costimulatory molecules, including PD-1, PD-L1, CTLA4, and CD80/CD86.^113^ In our studies, we observed that activated Vδ2 γδ T cells inhibited the proliferative response of CD4 αβ T cells in a CD86/CTLA4-dependent manner, as suggested by antibody blocking studies.^113^ An alternative mechanism implies upregulated cell surface expression of inhibitory PD-L1 on γδ T cells, which may then lead to the inhibition of αβ T cell activation. This has been shown for tumor-infiltrating non-Vγ9 γδ T cells in pancreatic adenocarcinoma.^103^ In a recent study, Schilbach and coworkers performed detailed studies to characterize the PD-L1-dependent suppressive activity of human Vδ2 γδ T cells. It was observed that the suppressive activity of activated Vδ2T cells on autologous αβ T cells was dependent on the signal strength of the TCR stimulation and enforced by IL-15, but it was independent of TGF-β (in line with the independence of FOXP3).^114^ The molecular mechanism of how γδ T cells suppress their neighbors is not precisely known. Conceivably, suppression might, in some instances, result from direct killing of αβ T cells by activated γδ T cells. Taken together, however, it is obvious that γδ T cells can acquire suppressive activity through a variety of different mechanisms. This would certainly be an unwanted effect in the context of cancer immunotherapy. Given the very limited success of clinical trials with unmodified γδ T cells or γδ T cell transfer/in vivo activation without additional “costimulatory” strategies (see below), the potential role of a suppressive function of γδ T cells in vivo requires careful consideration when attempting to harness their significant anticancer potential for cancer immunotherapy.

The activity of γδ T cells is also subject to regulation by the cellular context and the tumor micromilieu. γδ T cells are susceptible to inhibition by Treg cells^115^, which might be relevant in the context of cancer, as suggested in a study with hepatocellular carcinoma patients.^116^ Furthermore, the activity of γδ T cells is also regulated by multifaceted interactions with neutrophils.^117^ Neutrophils can inhibit the activation of γδ T cells, which has been mainly ascribed to neutrophil-derived reactive oxygen species (ROS).^118^ We also observed that neutrophils exposed to zoledronic acid (ZOL) inhibited the activation of Vδ2T cells, which was revealed when comparing the activation of γδ T cells present in Ficoll-Hypaque isolated PBMCs to total leukocytes following red blood cell lysis.^119^ Within PBMCs, monocytes incorporate aminobisphosphonates, such as ZOL, and generate the phosphoantigen IPP, which then activates Vδ2T cells.^120^ While neutrophils also take up ZOL, they fail to produce IPP^25^ but rather inhibit the activation of the γδ T cells. Neutrophil-derived ROS were also identified as a major inhibitory mechanism in our study; however, based on the effect of specific inhibitors, we also found arginase and serine proteases contributing to neutrophil-mediated γδ T cell suppression.^119^ Further studies identified elastase as the inhibitory serine protease of neutrophils.^121^ Interestingly, using phosphoantigen HMBPP rather than ZOL (used in our studies), Towstyka et al. observed that neutrophils and elastase actually costimulated IFN-γ production in anti-CD3 activated γδ T cells rather than exerting inhibitory effects.^122^ This apparent discrepancy illustrates the complexity of the relationship between neutrophils and γδ T cells, as different modes of activation of γδ T cells were applied: the indirect activation with ZOL used by us^119^ induced a neutrophil burst, which was not the case using direct stimulation of γδ T cells with pAg HMBPP^121^ or BrHPP (used by us as a control^119^). Further analyzing the regulatory interactions between neutrophils, tumor cells and γδ T cells in vitro, we also observed contrasting effects of neutrophils, depending on situational factors and the activation status of cells. While neutrophils present in leukocyte preparations inhibited the tumor killing capacity of γδ T cells within leukocytes following activation with ZOL, isolated neutrophils actually enhanced the killing capacity of short-term expanded γδ T cells by increasing their release of cytotoxic mediators.^123^ Seemingly opposing effects of reciprocal interactions between neutrophils and γδ T cells in the tumor microenvironment have also been observed in in vivo models. Tumor-associated neutrophils strongly inhibited IL-17 production by γδ T cells *via* the induction of oxidative stress, thereby exerting antitumoral activity in the tumor microenvironment.^124^ On the other hand, IL-17-producing γδ T cells were found to expand neutrophils in a granulocyte colony-stimulating factor (G-CSF)-dependent manner in a breast cancer model, which then actually suppressed CD8 T cell responses, thereby promoting metastasis formation.^80^

The tumor microenvironment can be rich in multiple factors that negatively impact T cells, including γδ T cells. Tumor cells and suppressive cells such as MDSCs frequently express ligands for inhibitory checkpoint receptors; for instance, PD-L1 and γδ T cells can express such receptors to varying degrees.^125^ Moreover, tumor cells themselves, tumor-associated macrophages, MDSCs and other cells within the microenvironment can produce a range of inhibitory molecules, including (but not limited to) TGF-β, IL-4, galectins, and indoleamine-2,3-dioxygenase (IDO), all of which may inhibit intratumoral γδ T cells from attacking the tumor.^126–130^ Arginase-I, an enzyme that suppresses both Vδ2 T cell cytotoxicity and IFN-γ production, can be produced by both tumor cells and MDSCs.^131^ Targeting such inhibitory pathways is an important aspect for improving the efficacy of T cell-based immunotherapies.

## Clinical studies with γδ T cells

Following the original observation by Kunzmann et al. of increased numbers of γδ T cells in the blood of patients with multiple myeloma treated with aminobisphosphonates for increased bone resorption,^132^ a number of small clinical studies have been performed to investigate the safety and efficacy of γδ T cell therapy in cancer patients. Two different approaches have been explored: (i) the in vivo activation of γδ T cells with aminobisphosphonates (usually ZOL) or (in rare instances) a phosphoantigen (BrHPP) plus low-dose IL-2 and (ii) the adoptive transfer of autologous or (rarely so far) allogeneic γδ T cells following in vitro expansion (again with ZOL or phosphoantigens). Such studies have been performed in various cancer diseases, including renal cell carcinoma, lung cancer, hepatocellular carcinoma, breast cancer, prostate cancer, and multiple myeloma. The general conclusions from those studies are as follows: (i) ZOL plus low-dose IL-2 application induces transient γδ T cell activation in vivo; (ii) adoptive transfer of expanded γδ T cells is safe, with usually only low levels of adverse events being observed; and (iii) even though clinical responses were recorded in most studies (ranging from partial remission and stable disease to complete remissions in exceptional cases), there is still—not surprisingly—room for substantial improvement. Several recent reviews have extensively documented past studies with in vivo activation or with adoptive transfer of γδ T cells, and the reader is referred to these publications for further information.^133–136^ In view of the HLA independence of γδ T cells, the application of allogeneic γδ T cells obtained from healthy donors could be considered. Haploidentical transplantation of hematopoietic stem cell (HSC) preparations depleted of αβ T cells and CD19^+^ B cells (thus containing NK cells and γδ T cells) for treatment of acute leukemia is now an established procedure,^137,138^ and haploidentical γδ T cells obtained by the depletion of CD4 and CD8 T cells from PBMCs have been infused into patients with advanced hematological malignancies.^139^ In this setting, γδ T cells are thought to contribute to the anti-leukemia effect.^140^ The limited experience thus far with adoptive transfer of allogeneic γδ T cells expanded from healthy donors in vitro has also shown a good safety profile in a case report in a patient with a solid tumor.^141^ While it needs to be considered that some γδ T cells might also display allo-reactivity,^140^ the application of γδ T cells freshly isolated or expanded from healthy donors rather than the patient’s own autologous γδ T cells might be a reasonable strategy for future application (see below). Some of the currently ongoing trials are mentioned in Table 1, while others are described in the literature.^136,142^

**Table 1:** Companies exploring concepts for γδ T cell based immunotherapy

Company	General strategy	Specific approach (if disclosed)	Web site
Adicet Bio, Inc.	Allogeneic gene-modified γδ T cells*	CAR or TCR-modified γδ T cells	https://www.adicetbio.com/
Cytomed Therapeutics	Allogeneic gene-modified γδ T cells*	CAR-modified (by mRNA electroporation) γδ T cells	https://www.cytomed.sg/
Editas medicine	Gene-modified γδ T cells	Not disclosed	https://www.editasmedicine.com
Gadeta	αβ T cells transduced with γδ TCR	High affinity Vγ9Vδ2 TCR (TEG)	https://www.gadeta.nl/
GammaCell Biotechnologies	Expansion of Vγ9Vδ2T cells		https://tracxn.com/d/companies/gammacelltech.com
GammaDelta Therapeutics	Allogeneic blood and skin-derived Vδ1 γδ T cells	Unmodified or CAR	https://gammadeltatx.com/
Immatics	Allogeneic gene-modified γδ T cells*	IMA301 Cancer testis antigen αβ TCR	https://immatics.com/
Incysus Therapeutics	Drug-resistant γδ T cells allogeneic γδ T cells	Allogeneic unmodified γδ T cells • Phase I study in leukemia patients undergoing hematopoietc stem cell transplantation Drug-resistant γδ T cells • Phase I study in glioblastoma	https://www.incysus.com/
Leucid Bio		CAR-modified γδ T cells	https://www.leucid.com
PhosphoGam Inc.	Allogeneic Vδ2 T cell transfer*	Off-the-shelf, selection of suitable donor-patient combinations	https://www.phosphogam.com
TC Biopharm	Allogeneic γδ T cell transfer*	• Unmodified or CAR modified TCB002 OmnImmune phase I (allogeneic unmodified γδ in AML)	https://www.tcbiopharm.com/
Adaptate Biotherapeutics	Modulate γd T cell activation in situ with antibodies		Spin-off of GammaDelta Therapeutics
American Gene Technologies	Transduce danger signal to tumor to activate γδ T cells in situ	3rd generation lentiviral vector, tumor cells then activate Vδ2 γδ T cells	https://www.americangene.com/
ImCheck Therapeutics	Targeting Vγ9Vδ2T cells in vivo	• Agonistic anti-BTN3A antibody: ICT01 (EVICTION Trial phase I/IIa) • Antagonistic anti-BTN3A antibody: ICT21 (autoimmune diseases)	https://www.imchecktherapeutics.com/
Lava Therapeutics	Targeting Vγ9Vδ2 T cells in vivo	Bispecific Vγ9Vδ2 T cell engagers targeting γδ T cells to tumor-expressed antigens	https://lavatherapeutics.com/
PureTech Health PLC	Targeting immunosuppressive Vδ1T cells in vivo	Human anti-Vδ1 antibody LYT-210	https://puretechhealth.com/

*Note*: Content is based on publicly disclosed information

*“off-the-shelf” products

## How to improve the in vitro expansion and effector activity of γδ T cells for adoptive transfer

Both Vδ1 and Vδ2 γδ T cells are in clinical development for adoptive cell therapy. Since mice do not express γδ TCRs homologous to human Vδ2 (i.e., mouse γδ T cells are not activated by phosphoantigens), immunodeficient or humanized mice transplanted with human tumors and γδ T cells are frequently used as a preclinical in vivo model.^143^ While both subsets can kill a broad range of malignant cells and show efficacy in xenograft models,^18,97,98,144–148^ they display different patterns of NK receptor and accessory molecule expression,^82^ and they also display related yet distinct cytotoxic hallmarks as revealed by recent single-cell RNAseq studies.^149^ The expansion of Vδ1 T cells with a specific protocol involving a two-step process with selected cytokines including IL-15 in the second step has been defined as DOT (“delta one T cells”).^97^ Other protocols for expanding highly cytolytic γδ T cells mainly of the Vδ1 variety utilized mitogen phytohemagglutinin (PHA) plus IL-7 stimulation^150^ or artificial antigen-presenting cells (APCs) expressing costimulatory molecules and CMV-pp65 antigens.^144^ Furthermore, polyclonal γδ T cells expressing various TCR VγVδ elements and broad cytotoxic activity against various tumor cells have also been generated in the presence of CD137L-expressing artificial APC and IL-2 plus IL-21.^98^ The most widely used protocol for selectively expanding Vδ2T cells relies on ZOL stimulation of PBMCs in the presence of IL-2,^100^ but similarly efficient Vδ2T cell activation in vitro can be achieved with synthetic pAgs such as BrHPP^20^ and HMBPP.^24,25^ After expansion for two weeks with continuous supply of IL-2 and careful control of the growth pattern, cell cultures starting from PBMCs (with 2–4% γδ T cells) can easily and reproducibly be expanded to contain 95% Vδ2T cells.^100^ Such short-term expanded Vδ2 T cell lines display strong cytotoxic activity against some tumor targets, but they show limited activity against others.^77,151^ Therefore, various optimization strategies have been investigated. ZOL has a rather narrow concentration range and is toxic at high concentrations.^25^ However, pulsing PBMCs with high concentrations (100 μM) of ZOL for four hours with subsequent washing steps was found to result in more efficient Vδ2 T cell expansion.^148^ Moreover, the use of bisphosphonate prodrugs rather than ZOL^152^ and the use of artificial APCs in addition to ZOL^153^ were also found to enhance the proliferative expansion and functional activity of Vδ2 γδ T cells. It should be mentioned that cellular cross-talk can significantly modulate the efficacy of Vδ2 T cell expansion and overall antitumor activity. In patients with multiple myeloma, ZOL-treated dendritic cells were superior to monocytes in expanding Vδ2T cells. In the additional presence of peptides of an HLA-A2-restricted tumor-associated antigen (survivin), this coculture system also amplified survivin-specific CD8 αβ T cells.^154^ Cytokines are also a critical component of in vitro γδ T cell expansion protocols. In this regard, common γ-chain family cytokines are pivotal for supporting proliferative expansion and cytotoxic effector function.^155^ Among those cytokines, IL-15 is particularly active in promoting cellular expansion and cytotoxic effector function.^148,155,156^ IL-15 has also been shown to upregulate the expression of CD56,^157^ which is known to be expressed on γδ T cells with potent cytotoxic activity.^51,158^ We also observed the upregulation of CD56 on Vδ2T cells by IL-15.^159^ Cytokines other than common γ-chain cytokines also modulate the cytotoxic potential of γδ T cells. Interestingly, we recently observed that TGF-β significantly increased the cytotoxic activity of isolated γδ T cells that were activated in vitro with pAgs in the presence of IL-2 and/or IL-15. TGF-β is usually considered an immunosuppressive cytokine, and TGF-β inhibits γδ T cell expansion if PBMCs are stimulated with ZOL. Mechanistically, we found that TGF-β strongly upregulated CD103 (the αE chain of the αEβ7 integrin), which is a receptor for E-cadherin frequently expressed on epithelial tumor cells. CD103-positive Vδ2T cells form prolonged synapses with E-cadherin-expressing tumor cells, and anti-CD103 antibodies reduced the killing capacity of TGF-β-expanded Vδ2T cells.^159^ The superior antitumor activity of tumor-specific CD8 αβ T cells expressing CD103 has been previously demonstrated.^160,161^ CD103 is a marker for resident memory cells, and CD103-positive TILs were associated with increased survival in high-grade serous ovarian cancer.^162^ CD103-expressing Vδ2T cells might migrate more efficiently into E-cadherin-positive tumor tissue. Therefore, it could be considered to induce CD103 expression on γδ T cells before adoptive transfer into patients with E-cadherin-expressing tumors. TGF-β-treated Vδ2T cells also potently produce IL-9, which might be an added benefit for adoptive transfer, given that IL-9 has multiple antitumor activities.^163^

Other strategies to improve proliferative expansion and effector functions of γδ T cells target metabolic pathways. In a recent study, it was reported that systemic β-adrenergic receptor activation, which was accomplished by dynamic physical exercise, mobilized γδ T cells to the blood and significantly augmented their subsequent in vitro expansion capacity and cytotoxic antitumor activity.^164^ A placebo-controlled crossover study applying adrenergic receptor inhibitors indicated that effects on the γδ T cell compartment were mediated by the β2—rather than the β1 adrenergic receptor.^164^

T cell activation and differentiation are also modified by vitamins. Vitamin C (Vit C, L-ascorbic acid), an essential vitamin, plays an important role in remodeling the epigenome^165^ and impacts T cell activation at multiple levels.^166^ The mechanistic basis of its action implies an interplay between antioxidant potential and (epi)genetic regulation of gene expression. We have investigated the effects of Vit C and the more stable phospho-modified L-ascorbic acid 2-phosphate (pVC) on the in vitro activation of Vδ2T cells. Proliferation and cytokine induction were significantly increased, and pVC strongly increased the proliferative expansion of short-term expanded Vδ2T cells following restimulation with pAg BrHPP, a condition that typically induces massive activation-induced cell death. Further studies showed that pVC reduced intracellular ROS levels and increased cell cycle progression and Ki-67 expression in surviving γδ T cells, thereby promoting the expansion of surviving cells rather than preventing cell death.^167^ Vδ2T cells expanded in the presence of pVC also displayed stronger cytotoxicity against tumor cells in vitro and were more active upon transfer into immunodeficient mice transplanted with a human lung tumor cell line (Yu et al., unpublished results). To conclude, the effect of Vit C on γδ T cell plasticity depends on the overall environmental signals.^166,167^ As discussed above, Vit C actually conveys a regulatory phenotype and induces *FOXP3* hypomethylation in the additional presence of TGF-β.^112^ However, in the absence of TGF-β during the expansion phase, Vit C substantially enhances effector functions desired in the context of cancer immunotherapy. This may also include the potent production of IL-13,^167^ which is known to contribute to antitumor immunity.^168^ Therefore, we suggest including Vit C/pVC in in vitro γδ T cell expansion protocols for adoptive immunotherapy.

Despite the many strategies briefly summarized here to enhance the in vitro expansion and functionality of expanded γδ T cells, the question remains whether the expanded γδ T cells are sufficiently effective to induce a clinically important response in patients. This issue is mainly related to clonal heterogeneity even among a defined cell population such as Vγ9Vδ2T cells.^37^ Therefore, it is important to consider how γδ T cells can be engineered for optimal functionality.

## Design your desired γδ T cells

In recent years, genetic engineering of αβ T cells has been widely explored as a tool to improve cancer immunotherapy.^169^ Chimeric antigen receptor-modified T (CAR-T) cells that express CAR molecules that target surface antigens on tumor cells have revolutionized the treatment of B-cell malignancies but have yet to achieve the same level of success for solid tumors.^170^ γδ T cells are interesting recipient cells for CAR constructs as the transfection should result in effector cells with two-fold antitumor activity, e.g., (i) through the endogenous γδ TCRs and (ii) through the CAR specificity.^171^ In fact, CAR-transduced Vδ2T cells showed enhanced cytotoxicity towards relevant tumor target cells.^172^ Activated Vδ2T cells can act as APCs and cross-present tumor-derived peptides to CD8 αβ T cells upon the killing of tumor cells.^173^ Importantly, the ability for cross-presentation of tumor antigens to αβ T cells was preserved in CAR-transduced Vδ2T cells.^172^ γδ T cells can also be transfected with tumor antigen-specific αβ TCRs, such as HLA-A2-restricted melanoma-related gp100-specific αβ TCRs, again resulting in effector cells with dual antitumor specificity.^174^ An alternative and different approach is to transduce the αβ T cells of cancer patients with high-affinity Vγ9Vδ2 TCRs, termed T cells engineered with defined γδ TCRs (TEGs).^175^ This is based on the fact that not all Vγ9Vδ2 TCRs display equally high affinity for pAgs and, thus, tumor cell recognition. An added advantage of the strategy is that CD8, as well as CD4 αβ T cells are transduced with the selected Vγ9Vδ2 TCRs, thereby enabling CD4 T cells to exert helper functions such as the induction of dendritic cell maturation.^135^ TEGs expressing a high-affinity Vγ9Vδ2TCR have been manufactured under GMP conditions, and a clinical trial exploring safety and tolerability has been initiated (https://www.trialregister.nl/trial/6357).^176^ Alternatively, NK cells might be suitable recipient cells for the transduction of selected high-affinity γδ TCRs. Such an approach has been recently reported for the generation of anti-CD19 CAR-expressing NK cells, which mediated clinical responses in patients with relapsed or refractory CD19-expressing malignancies.^177^ The combination of intrinsic NK cell properties with a high-affinity antitumor-directed γδ TCR might reveal significant synergistic potential.

Genetic engineering is also used to render γδ T cells resistant to chemotherapeutic drugs used to treat cancer patients. Glioblastoma multiforme is a cancer in which local instillation of γδ T cells following surgery is considered a promising immunotherapeutic approach and has been demonstrated to be effective in a preclinical model.^147^ Local administration of γδ T cells appears feasible from a safety perspective, as allogeneic γδ T cells do not injure normal brain tissue.^178^ Lamb and colleagues designed a lentiviral vector expression system encoding a DNA repair enzyme. The transduction of γδ T cells conferred resistance to temozolomide, the widely used standard chemotherapeutic drug to treat glioblastoma patients.^179^ Temozolomide resistance did not alter the expansion capacity and cytotoxic activity of γδ T cells, suggesting that drug-resistant γδ T cells might be applicable in a clinical setting. A phase I study investigating the safety and tolerability of drug-resistant γδ T cell infusion is currently ongoing (https://clinicaltrials.gov/ct2/show/NCT04165941).

## How to activate and target γδ T cells in vivo

As summarized in recent reviews, ZOL in combination with low-dose IL-2 has been used in an attempt to activate (and possibly expand) tumor-reactive Vδ2T cells in vivo.^133–136^ While the treatment regimens varied in these studies, an increase in circulating γδ T cells was usually observed within one week.^180–182^ In addition, ZOL treatment substantially shifted the phenotype of Vδ2T cells to effector memory (T_EM_) cells.^183,184^ However, the expansion of γδ T cells in the peripheral blood was not sustained. In fact, repeated application of ZOL infusions resulted in a progressive decline in γδ T cells,^180,184^ an observation that we also made in patients with osteoporosis newly prescribed i.v. bisphosphonate therapy.^185^ Haploidentical HSC transplantation of αβ T cell/CD19 B-cell-depleted cells is now an established therapeutic procedure for certain blood malignancies. γδ T cells comprise the major CD3^+^ T cell population during reconstitution early after transplantation. In addition to their potential anti-leukemia effect, γδ T cells are also important for the control of Epstein-Barr virus (EBV) reactivation and related lymphoproliferative diseases and for the containment of CMV reactivation after HSC transplantation. In fact, the inflammatory milieu might be important for early γδ T cell control.^186^ Patients undergoing haploidentical HSC transplantation are routinely treated with immunosuppressive drugs such as mycophenolate mofetil (MMF) to prevent graft-versus-host disease (GvHD). Early reduction of MMF was associated with improved Vδ2 T cell recovery and decreased EBV reactivation.^187^ ZOL has also been used for in vivo activation of γδ T cells after αβ T cell/CD19 B-cell-depleted haploidentical HSC transplantation in children with acute leukemia. Following repeated ZOL administration, the γδ T cell numbers declined more than in the control group; however, γδ T cells differentiated into T_EM_ cells and were more cytotoxic in the ZOL-treated cohort. Despite decreasing γδ T cell counts, the incidence of GvHD and transplant-related mortality were lower in patients receiving ≥3 ZOL infusions.^188,189^ These studies suggest that in vivo activation of γδ T cells with ZOL is a useful treatment option after HSC transplantation with αβ T cell/CD19 B-cell-depleted stem cell preparations. However, even though objective responses were observed in some patients (see the abovementioned reviews), continuous therapy with ZOL and low-dose IL-2 is not an efficient option for γδ T cell immunotherapy of patients with solid cancers (also in view of the effect of low-dose IL-2 in Treg activation^190^) but might be useful as a transient procedure together with other strategies. In vivo activation and expansion of Vδ2T cells has also been achieved with i.v. infusion of pAg BrHPP (Phosphostim) together with s.c. low-dose IL-2.^191^ Originally developed as a γδ T cell immunotherapy, this strategy was abandoned as it failed in further clinical trials.

As mentioned earlier, agonistic mAbs directed against BTN3A1/CD277, such as clone 20.1, are very potent and selective activators of Vδ2T cells.^25,31^ BTN3A1 is expressed on tumor cells,^192^ and sensitizing tumor cells with an anti-BTN3A1 mAb drastically increases sensitivity to γδ T cell killing.^31^ Therefore, a novel strategy for in vivo activation of tumor-reactive Vδ2 T cells is the therapeutic application of a humanized anti-BTN3A1 mAb. In fact, a phase I/IIa clinical trial with such an antibody (ICT01) has just started (https://www.clinicaltrials.gov/ct2/show/NCT04243499). However, given that BTN3A1 is also widely expressed on normal cells, a concern is that normal cells—and not just transformed cells—could also be sensed and killed after binding of an anti-BTN3A1 mAb. The difference might be related to much more efficient BTN3A1 clustering on tumor cells than on normal cells,^192,193^ but potential adverse effects will need to be closely monitored in the ongoing trial. Based on the wide distribution of the target molecule, the risk of inducing autoimmunity is a potential concern.

Another strategy to specifically target γδ T cells to cancer cells in vivo is bispecific antibody constructs that cross-link the TCRs on γδ T cells with tumor surface antigens. Using Her2-neu as a model antigen, it was shown that a bispecific Her2-Vγ9 antibody construct (designed in a “tribody” format with two anti-Her2 single-chain variable fragments [scFvs] linked to the Fab fragment of an anti-Vγ9 mAb)^194,195^ efficiently triggered the killing of Her2-expressing tumor cells by short-term expanded Vγ9Vδ2 T cell lines.^194,195^ Such antibody constructs also efficiently triggered the cytotoxic activity of γδ TILs against autologous cancer cells.^77^ There are multiple strategies for the rational design of even smaller molecules, such as single-domain nanobodies with low immunogenicity that may increase the likelihood of accumulating around the actual tumor site for the activation of tumor-resident γδ T cells.^196,197^ In preclinical proof-of-principle studies, the therapeutic efficacy of such anti-EGFR-Vγ9 nanobodies has been demonstrated,^198^ and bispecific γδ T cell engagers are in development for targeting γδ T cells in cancer patients. Lava Therapeutics’ first bispecific γδ T cell engager (see Table 1) against a novel target is entering clinical trials with first patients with a hematological indication anticipated for the end of 2020 (P. Parren, personal communication).

Taken together, there are various strategies available for the in vivo activation and targeting of γδ T cells as opposed to the adoptive transfer of in vitro expanded γδ T cells. Clinical studies will reveal the respective advantages and disadvantages. Overall, however, we believe that γδ T cell-targeted immunotherapy will need to be combined with other approaches to optimally enhance efficacy. Some possible strategies are discussed in the following section.

## Combination matters: how to improve γδ T cell therapy

Successful T cell immunotherapy of cancer requires the optimization of several key issues: (i) tumor-targeted effector activity of T cells; (ii) the sensitivity of cancer cells to T cell attack; (iii) the infiltration of T cells into the tumor tissue (particularly important in the case of “cold” tumors^199^); and (iv) overriding the immunosuppressive tumor micromilieu. In principle, this applies both to in vivo activation and to adoptive transfer of γδ T cells, as discussed above.

### Antibodies and checkpoint inhibitors

A substantial proportion of γδ T cells express the low-affinity Fc receptor for IgG (FcγRIII; CD16), and CD16-dependent antibody-dependent cellular cytotoxicity (ADCC) can be mediated by Vδ2T cells. Therefore, the combination of clinically used therapeutic antibodies with adoptive Vδ2 T cell transfer might enhance the efficacy.^200,201^ Further, γδ T cell activity is also regulated by checkpoint receptors. Vδ2T cells transiently upregulate PD-1 expression upon activation.^113,202,203^ PD-1 blockade with antibodies such as pembrolizumab does not significantly modulate the killing capacity^202,203^ but enhances IFN-γ production in ZOL-activated Vδ2T cells.^203^ Depending on the status of PD-1 expression on γδ T cells, combination with pembrolizumab might be envisaged for γδ T cell therapy, similar to what has recently been shown for allogeneic NK cell therapy.^204^ Another example of an inhibitory receptor is NKG2A, which can be expressed together with CD94 on γδ T cells.^205^ NKG2A has recently been identified as a novel checkpoint inhibitor, and the humanized anti-NKG2A mAb monalizumab unleashes antitumor immunity mediated by CD8 T cells and NK cells.^206,207^ While the role of γδ T cells has not been specifically addressed in these studies, it will be interesting to investigate the possible effects of monalizumab on the activation and effector function of NKG2A-expressing γδ T cells.

### Chemotherapy and epigenetic drugs

Several studies have demonstrated that standard chemotherapeutic drugs or kinase inhibitors frequently increase the cancer cell susceptibility to γδ T cell killing, for instance, for colon cancer,^208^ glioblastoma,^209^ ovarian cancer,^210^ or chronic lymphocytic leukemia.^211^ Thus, relevant preclinical model systems suggest synergistic effects of combined chemotherapy and adoptive transfer of allogeneic Vδ2T cells.^210^ A large number of substances have been shown to epigenetically modify gene expression at the level of DNA methylation and histone modification, and several drugs, including the histone deacetylase inhibitor valproic acid (VPA) and the DNA demethylating agent decitabine, are clinically used.^212^ VPA was shown to synergize with ZOL in enhancing γδ T cell cytotoxicity at the level of pAg production^213^ but also affected the interaction between γδ T cells and tumor cells at the level of the NKG2D receptor/ligand axis.^214^ By contrast, decitabine increased the sensitivity of osteosarcoma cells to Vδ2 T cell killing by upregulating the cell surface expression of NKG2D ligands.^215^ Overall, epigenetic drugs offer interesting perspectives for combination with γδ T cell immunotherapies.^216^

### Accessibility of solid tumors

Adoptively transferred or endogenous γδ T cells need to infiltrate into the tumor to exert their antitumor activity. It has been demonstrated that local low-dose gamma irradiation can cause the normalization of aberrant vasculature and efficient recruitment of tumor-specific T cells,^217^ and this may also apply to enhancing γδ T cell migration into the tumor. The remodeling of the extracellular matrix by an inhibitor of hyaluronan synthesis has been shown to enhance γδ T cell cytotoxicity against pancreatic adenocarcinoma cells in vitro and to promote the infiltration of γδ T cells into tumor tissue, thereby suppressing tumor growth in xenografted mice.^218^ Another strategy to enhance antitumor immunity is the activation of innate immunity by ligands for Toll-like receptors (TLRs) or cyclic GMP-AMP synthase (cGAS)/stimulator of interferon genes (STING). The modulation of tumorigenesis by TLR/STING ligands is complex and context-dependent. In fact, it can also promote tumor development under certain conditions.^219^ Similarly, the modulation of γδ T cell activation by specific innate receptors, such as TLR8, also depends on the cellular context and functional outcome parameters. We recently observed strong and monocyte-dependent costimulation of IFN-γ production in Vδ2T cells by TLR8 ligands, whereas proliferative expansion of Vδ2T cells in response to pAgs was simultaneously inhibited.^220^ Among the many translational perspectives, TLR/STING ligands are considered adjuvants for cancer vaccines.^219,221^ The intratumoral application of TLR^222,223^ and STING ligands^224^ may additionally be used to increase the inflammatory condition of the tumor in situ, thereby allowing more efficient migration of T cells, including γδ T cells, into the tumor microenvironment.

### Reversal of the immunosuppressive tumor microenvironment

Tumor cells, tumor-associated macrophages, MDSCs, and other tumor stromal cells can work together to potently suppress intratumoral immune responses. Tumor-intrinsic mechanisms that have been identified to impair γδ T cell attack include the release of large amounts of prostaglandin E2 by tumor cells with strong expression of cyclooxygenase-2 (COX-2),^151^ the activity of indoleamine-2,3-dioxygenase (IDO) and its metabolite kynurenine,^225^ the release of galectin-3,^226^ and the hypoxic tumor microenvironment.^227^ Inhibitors for the respective pathways can enhance tumor killing by Vδ2T cells in vitro, and it seems reasonable to propose that these strategies can also work in vivo, given the availability of approved drugs such as COX2 inhibitors. In addition to galectin-3, other tumor-expressed galectins, such as galectin-9, also suppress T cell activation.^228^ The galectin-9 receptor, namely, T cell immunoglobulin domain and mucin domain 3 (Tim-3), on Vδ2T cells has recently been shown to suppress their killing capacity by reducing perforin and granzyme B expression.^229^ A human anti-galectin-9 antibody has been developed for clinical application,^230^ and its combined use with γδ T cell immunotherapy may be synergistic. Another important aspect is the hostile metabolic environment for T cells within the tumor, which includes hypoxia, glucose depletion, and lactate accumulation. There exist multiple strategies to optimize T cell metabolism to improve cellular immunotherapy,^231^ and it will be important to consider these for harnessing the full potential of γδ T cell immunotherapy.

A summary of current strategies to enhance cellular expansion/effector activity in vitro and the clinical efficacy of γδ T cell therapy in vivo is illustrated in Fig. 3.

## Future directions

As discussed in this article, there are multiple fronts for future development and optimization to bring to fruition the promise of γδ T cells into clinically effective cellular therapeutics. Here, we highlight just a few of the many noteworthy advances.

### Preclinical evaluation

The only relevant animal model to evaluate the in vivo activity of human Vδ2 γδ T cells is nonhuman primates, which also harbor Vγ9Vδ2T cells. Given the lack of readily available appropriate tumor models and the exorbitant costs, it is not feasible to properly evaluate antitumor activity of Vδ2T cells in nonhuman primates. Conventional mice harbor neither homologous γδ TCRs nor BTN2A/3A-homologous butyrophilins; therefore, these cannot be used to address the functionality of Vδ2 γδ T cells. As a result, immunodeficient mice or different types of humanized mice are routinely used to test for antitumor activity of human γδ T cells, but again, this approach does not allow the extrapolation of predictions as to safety and efficacy when applied to humans.^143,146^ We suggest that more efforts should be devoted to the development of advanced in vitro models, such as three-dimensional spheroid cultures, which allow better predictions than regular two-dimensional tumor γδ T cell cocultures.^232^

### Off-the-shelf γδ T cell products for immunotherapy

As discussed, there is good evidence that the application of allogeneic rather than autologous γδ T cells might be feasible. This opens up the perspective that γδ T cells from healthy blood donors can be manufactured under GMP conditions and stored until required for adoptive immunotherapy of cancer patients. Several companies are pursuing this strategy, as summarized in Table 1.

### Biomarkers for tumor susceptibility

Not all transformed cells are equally susceptible to γδ T cell killing. Whenever possible, patients who are being considered for γδ T cell immunotherapy should be selected on the basis of predictive biomarkers. In the case of acute myeloid leukemia (AML), the expression of the NKG2D ligand ULBP1 on AML blasts may be a predictive biomarker for efficacy.^233^ Conversely, off-the-shelf produced GMP-expanded γδ T cell products generated from different blood donors might not be equally effective against various tumor entities. Pretesting against a panel of tumor targets could help select the most suitable product for each patient.

### Coactivation of innate immunity

Above, we discussed the role of TLR/STING activation of innate immunity to support the antitumor activity of γδ T cells. There are multiple additional pathways in the innate immune system that need to be investigated for their potential ability to optimally harness and support the effector functions of tumor-reactive γδ T cells.^234^ An exciting new perspective arises from recent evidence that organ-specific activation of innate immunity can be triggered by inactivated microbes that are endogenous within specific organ sites. Based on this concept, microbial preparations, called site-specific immunomodulators (SSIs), derived from *Klebsiella variicola*, *Escherichia coli*, and *Staphylococcus aureus*, have been developed and shown in mouse models to have organ-specific effects on the lung, intestine or skin and can stimulate organ-specific antitumor responses.^235^ SSIs can also enhance the efficacy of adoptively transferred antitumor T cells by supporting the infiltration of T cells into the tumor microenvironment and increasing tumor immunogenicity.^236^ Therefore, it is anticipated that SSIs would provide important additional activation of tumor-reactive γδ T cells in an organ-specific manner, a concept that we believe should be explored in the future.

### High-dose Vit C

The results from our studies have proven that γδ T cell activation and cytotoxicity in vitro can be enhanced by Vit C. These results raise the question of whether Vit C could also enhance γδ T cell activity in vivo. Recently, it was shown that high-dose Vit C increased the efficacy of cancer immunotherapy in various mouse models by enhancing the cytotoxic activity of CD8 T cells and by cooperating with immune checkpoint inhibition.^237^ In fact, high-dose i.v. application of Vit C in cancer patients is used in some centers, and studies have shown a very good safety profile of high-dose Vit C therapy.^238^ Therefore, high-dose i.v. Vit C therapy might also be considered when thinking of ways to enhance the efficacy of γδ T cell therapy.

## Concluding remarks

Even though there is now fierce competition for determining which cells should be invested in and taken forward for cancer immunotherapy, we believe that the unique properties of γδ T cells put them at the forefront. Support for this idea is evident in the recent burst of interest from small and not-so-small biotech companies exploring the immunotherapeutic potential of γδ T cells, as summarized in Table 1. After 35 years of research to understand the peculiarities of γδ T cells, it is rewarding to witness the current multiple activities to bring these cells into clinical application to treat cancer patients.
