# Correction to: Cancer immunotherapy with γδ T cells: many paths ahead of us

**DOI:** 10.1038/s41423-020-00537-z

**Published:** 2020-09-01

**Authors:** Dieter Kabelitz, Ruben Serrano, Léonce Kouakanou, Christian Peters, Shirin Kalyan

**Affiliations:** 1 Institute of Immunology, Christian-Albrechts University of Kiel and University Hospital Schleswig-Holstein Campus Kiel, D-24105 Kiel, Germany; 2 Faculty of Medicine, University of British Columbia, Vancouver, BC V6T 1Z4 Canada

**Keywords:** Immunosurveillance, Immunology

Correction to: *Cellular & Molecular Immunology* 10.1038/s41423-020-0504-x, published online 22 July 2020

The article “Cancer immunotherapy with γδ T cells: many paths ahead of us”, written by Dieter Kabelitz, Ruben Serrano, Léonce Kouakanou, Christian Peters and Shirin Kalyan, was originally published electronically on the publisher’s internet portal on 22 July 2020 without open access. With the author(s)’ decision to opt for Open Choice the copyright of the article changed on 3 August 2020 to © The Author(s) 2020 and the article is forthwith distributed under a Creative Commons Attribution 4.0 International License, which permits use, sharing, adaptation, distribution and reproduction in any medium or format, as long as you give appropriate credit to the original author(s) and the source, provide a link to the Creative Commons licence, and indicate if changes were made. The images or other third party material in this article are included in the article’s Creative Commons licence, unless indicated otherwise in a credit line to the material. If material is not included in the article’s Creative Commons licence and your intended use is not permitted by statutory regulation or exceeds the permitted use, you will need to obtain permission directly from the copyright holder. To view a copy of this licence, visit http://creativecommons.org/licenses/by/4.0.

